# Test-retest reliability of spatial navigation in adults at-risk of Alzheimer’s disease

**DOI:** 10.1371/journal.pone.0239077

**Published:** 2020-09-22

**Authors:** Gillian Coughlan, Vaisakh Puthusseryppady, Ellen Lowry, Rachel Gillings, Hugo Spiers, Anne-Marie Minihane, Michael Hornberger

**Affiliations:** 1 Norwich Medical School, University of East Anglia, Norwich, United Kingdom; 2 Department of Psychology, University of East Anglia, Norwich, United Kingdom; 3 Department of Experimental Psychology, Institute of Behavioural Neuroscience, University College London, London, United Kingdom; Nathan S Kline Institute, UNITED STATES

## Abstract

The Virtual Supermarket Task (VST) and Sea Hero Quest detect high-genetic-risk Alzheimer`s disease (AD). We aimed to determine their test-retest reliability in a preclinical AD population. Over two time points, separated by an 18-month period, 59 cognitively healthy individuals underwent a neuropsychological and spatial navigation assessment. At baseline, participants were classified as low-genetic-risk of AD or high-genetic-risk of AD. We calculated two-way mixed effects intraclass correlation coefficients (ICC) for task parameters and used repeated measures ANOVAS to determine whether genetic risk or sex contributed to test-retest variability. The egocentric parameter of the VST measure showed the highest test–retest reliability (ICC = .72), followed by the SHQ distance travelled parameter (ICC = .50). Post hoc longitudinal analysis showed that boundary-based navigation predicts worsening episodic memory concerns in high-risk (F = 5.01, *P =* 0.03), but in not low-risk, AD candidates. The VST and the Sea Hero Quest produced parameters with acceptable test-retest reliability. Further research in larger sample sizes is desirable.

## Introduction

Spatial navigation shows promise as an outcome measure for preclinical Alzheimer disease (AD) in drug treatment trials, but it’s test-retest reliability is not clear [1–4]. Drug development for AD has been plagued by the failure of cognitive outcomes measures to detect treatment response due to i) insufficient sensitivity and specificity for preclinical neuropathology and/or ii) poor test-retest reliability, both of which can mask neural response to treatment [5–8].

Novel spatial navigation measures, the Virtual Supermarket Task (VST) and Sea Hero Quest (SHQ) identify cognitive changes due to functional neural abnormalities within the spatial navigation network (particularly the entorhinal cortex and hippocampus) in at-genetic-risk AD, making them sensitive and specific measures for preclinical AD disease [2, 9–11]. Until now however, spatial navigation studies have overlooked the need to establish the test-retest reliability of these measures in preclinical AD populations, opting to focus on cross-sectional group comparisons or self-report scales [12], with one exception [13].

The importance of rest–retest reliability relies in its ability to determine the degree to which baseline and follow-up assessments produce consistent results [14–16] and it is often confounded by practice effects due to repeated exposure to the same trials [17, 18]. For example, participants may learn to apply strategies at retest that improve their navigation accuracy or efficiency, and in turn reduce test–retest reliability by lowering the sensitivity of that test measure to signal the degree of preclinical neuropathology [19, 20]. The use of alternate forms of the same test measure is often recommended but can still be vulnerable to biases due to problem-solving strategies developed at baseline.

Having previously determined the diagnostic utility of VST and SHQ in at-genetic-risk AD, we aimed to establish the test-retest reliability of these two spatial navigation tasks. The VST was originally developed to distinguish AD from other dementias [21], while SHQ was designed to measure navigation ability on a global scale (see Coutrot et al. [22] for more details). We also included the Four Mountains test, a well-established measure of spatial memory in mild cognitive impairment and Alzheimer’s disease, as a standard measure to compare the reliability of the novel spatial navigation tasks [23, 24]. We predicted that demographic and genetic factors, such as sex and the apolipoprotein allele ε4 (*APOE*) gene, may influence test-retest reliability, similar to many gold standard diagnostic tests [7, 25]. We predicted that some scoring parameters in each spatial navigation measure would be more reliable than others, meaning that some measures may be less vulnerable to practice or novelty effects. For example, the response format for one VST parameter was altered at retest in an attempt to increase the parameter’s sensitivity to spatial memory impairments. We predicted this parameter would exhibit low reliability over timepoints. Finally, we examined if the apolipoprotein (APOE) ε4 sensitive navigation parameters predict change in subjective cognitive concerns or neuropsychological performance at retest. In particular, subjective cognitive concerns are typically used characterise preclinical AD [26] The baseline findings for this study are published elsewhere [9, 10].

## Materials and methods

### Participants

In this cohort study, low-risk (ε3ε3 carriers) and high-risk (ε3ε4 carriers) were assessed at enrolment and 18 months later. All participants were pre-screened for a history of psychiatric or neurological disease, history of substance dependence disorder, or any significant relevant comorbidity. All participants had normal or corrected-to-normal vision. Family history of AD and history of antidepressant treatment with serotonin reuptake inhibitor drugs was retrospectivity obtained. Saliva samples were collected from those who passed this screening, and APOE genotype status was determined. At baseline (May-December 2017) and follow-up (September 2018-January 2019), participants underwent a neuropsychological examination including the cognitive change index (CCI) and a novel cognition test battery including the VST, SHQ and the four mountains test [9, 22, 23]. At the retest analysis, we included participants who participated in both assessments (baseline [T1] n = 64; ε3ε3 = 33, ε3ε4 = 31; retest [T2]: n = 59; ε3ε3 = 32, ε3ε4 = 28), which equals an attrition rate of 3.25% (5 dropouts). Reasons for dropout included ‘personal time constraints’ and ‘lack of sustained interest’. One other participant developed lupus (a systemic autoimmune disease) over the study period and was excluded. Mean age of participants at baseline was 61.9 ± 6.7 years and at retest was 64.08 ± 5.9 years. The age range of the sample was 51–72 years. The average follow-up duration was 18 months ± 0.4 months (see Table 1 for age, sex educational background of the sample). The SHQ platform was removed for two weeks in December 2018, which resulted in less SHQ data being collected at retest (T2) compared to baseline (T1). As a result, the test-retest reliability of SHQ included a reduced sample size (n = 44).

**Table 1:** Demographic characteristic of the sample.

	Mean (SD)		
		*APOE genotype*	
Demographic characteristic	Total (n = 59)	ε3ε4 carriers (n = 27)	ε3ε3 carriers (n = 32)
** Age (T2), y**	64.08 (5.9)	63.74 (6.4)	64.38 (5.6)
** Sex (male/female)**	26/33	8/19	18/14
** Education, y**	14.4 (5.4)	14.5 (2.9)	14.4 (3.6)

Data are presented as mean (SD).

### Procedure

**Fig 1:**
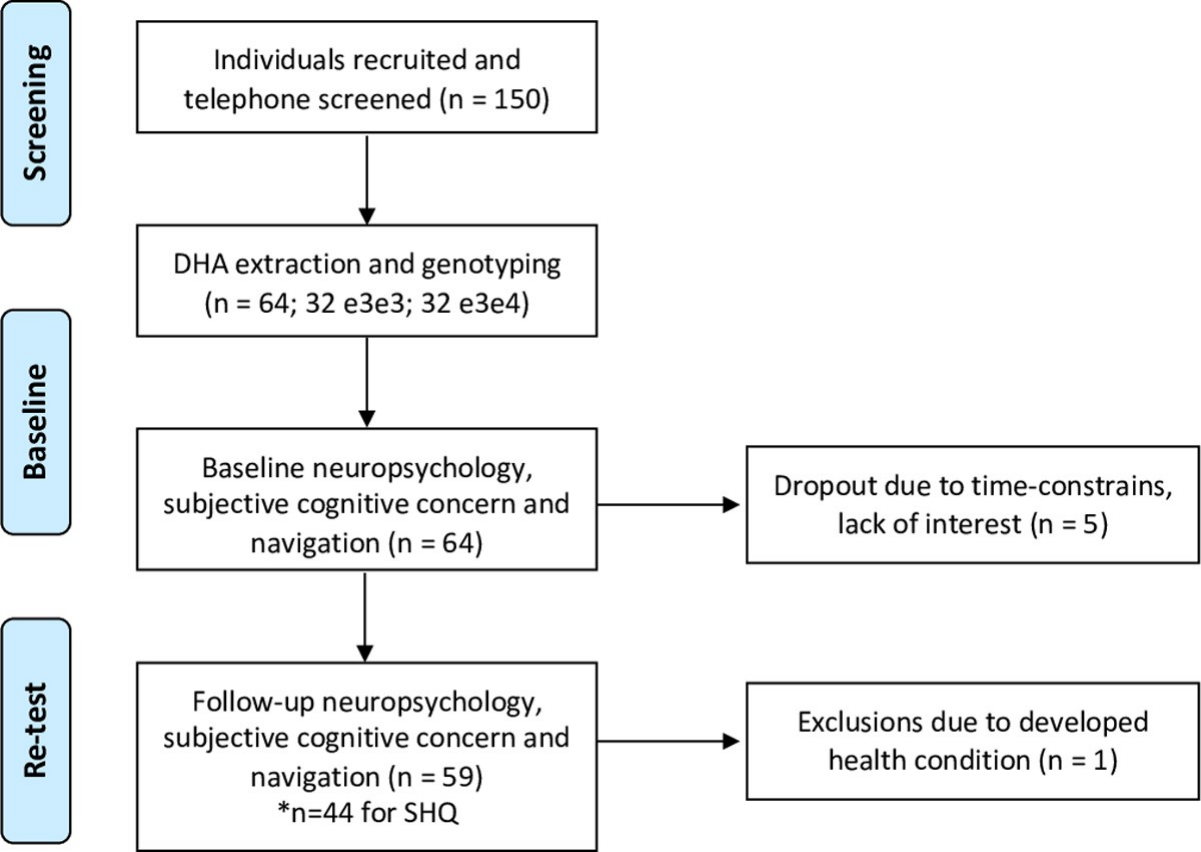
Longitudinal design at baseline (T1) and retest (T2). *Note a sub-set of individuals did not complete SHQ at both timepoints, reducing the sample size (n = 44).

The Faculty of Medicine and Health Sciences Ethics Committee at the University of East Anglia approved the experimental procedures (reference FMH/2016/2017-11) and written consent was obtained from all participants. Telephone screening and *APOE* genotyping was completed by the study team before baseline cognitive assessments commenced. At baseline and follow-up, participants completed a two-hour cognitive *testing* session. Cognitive testing took place in a quiet laboratory setting and was conducted by an experienced tester at both timepoints to aid retest reliability, as recommended by Aarts and colleagues (2015) [27]. See Fig 1 for a visual representation of the study design. The *APOE* genotyping method can be found here [9].

#### APOE genotyping

DNA was collected using a Darcon tip buccal swab (LE11 5RG; Fisher Scientific). Buccal swabs were refrigerated at 2–4°C until DNA was extracted using the QIAGEN QIAamp DNA Mini Kit (M15 6SH; QIAGEN). DNA was quantified by analyzing 2-μL aliquots of each extraction on a QUBIT 3.0 fluorometer (LE11 5RG; Fisher Scientific). Successful DNA extractions were confirmed by the presence of a DNA concentration of 1.5 μg or higher per 100 μg of AE buffer as indicated on the QUBIT reading. PCR amplification and plate read analysis was performed using Applied Biosystems 7500 Fast Real-Time PCR System (TN23 4FD; Thermo Fisher Scientific). TaqMan Genotyping Master Mix was mixed with two single-nucleotide polymorphisms of APOE (rs429358 at codon 112 and rs7412 at codon 158). These two single-nucleotide polymorphisms determine the genotype of APOE2, Ε3, and Ε4 (2007; Applied Biosystems).

### Measures

#### Neuropsychological and subjective cognition assessment

A neuropsychological assessment was included to investigate change in global cognitive function and self-report cognitive function across the timepoints [28]. The assessment consisted of the Cognitive Change Index (CCI); a measure of self-report episodic memory and executive function ability [29]. The assessment also included the Addenbrooke’s Cognitive Examination—III (ACE) version B at baseline and version C at retest. Similarly, the Rey Osterrieth Complex Figure test (ROCF) was administered at baseline and the Taylor Complex Figure task was administered at retest (see Table 2) [30, 31]. Alternative task versions were administered at timepoints to reduce retest effects.

**Table 2:** Neuropsychological performance from baseline (T1) and retest (T2) between genetic groups.

Measure	Variable	Mean T1	Mean T2	Δ	*p* value
**ACE**	Total ε3ε3	94.67 ± 3.67	93.70 ± 4.88	-.97 ± 5.33	0.06 (t = 1.903)
	Total ε3ε4	92.96 ± 3.82	94.37 ± 2.31	1.41 ± 3.35	
	Memory ε3ε3	24.70 ± 1.92	24.97 ± 1.43	.27 ± 2.13	0.03 (t = 2.120)
	Memory ε3ε4	23.70 ± 1.66	25.00 ± 1.07	1.26 ± 1.75	
	Visuospatial ε3ε3	14.93 ± 1.05	14.20 ± 1.32	-.73 ± 1.34	0.60 (t = 0.523)
	Visuospatial ε3ε4	14.85 ± 1.21	14.15 ± .94	-.70 ± .99	
**ROCF**	Copy 3ε3	33.23 ± 2.77	32.75 ± 2.84	-.38 ± 2.91	0.88 (t = 0.145)
	Copy ε3ε4	32.28 ± 2.62	32.15 ± 2.57	-.18 ± 2.87	
	Recall ε3ε3	20.51 ± 6.32	21.83 ± 5.34	1.06 ± 4.73	0.13 (t = 1.516)
	Recall ε3ε4	18.15 ± 6.11	21.50 ± 5.02	2.60 ± 7.68	
**CCI**	Episodic ε3ε3	19.28 ± 6.46	18.40 ± 6.29	.39 ± 4.12	0.38 (t = -0.87)
	Episodic ε3ε4	21.48 ± 6.76	22.36 ± 6.44	-.96 ± 5.42	
	EF ε3ε3	11.47 ± 4.53	10.45 ± 3.77	.64 ± 3.12	0.02 (t = -2.244)
	EF ε3ε4	11.56 ± 3.75	12.75 ± 4.37	-1.22 + 2.53	

ACE = The Addenbrooke’s cognitive examination; ROCF = Rey-Osterrieth complex figure test; CCI = Cognitive change index; EF; Executive function; T1 = Baseline; T2 = retest; Δ change/difference = T2 value—T1 value; *p* value = significant change between genetic groups.

#### Spatial navigation performance assessment

The VST, SHQ and the Four Mountains test have previously demonstrated feasibility in clinical populations. A list of seven scoring parameters in each of the three navigation measures can be found in Table 3.

**Table 3:** Intra-class correlations coefficients, mean change, and coefficient of variation.

Task parameters	ICC (95% CI)	MC (95% CI)	CoV%
**VST**			
Egocentric	**.72 (**.530–.838)	-0.857 (-1.67 –-.04)	18.90
Map drop	-	-	-
Heading	**.50 (.**148–.710)	0.0818 (-0.704–0.867)	15.43
CNP	.27 (-.26–.576)	-0.050 (-0.107–0.006)	26.35
**SHQ**			
Distance	**.50 (**.058–.719)	-0.275 (-0.560–0.0096)	12.26
Duration	.48 (.052–.718)	-0.636 (-1.249–0.0240)	19.27
**4MT**			
Total	**.50**. (153–.703)	0.732 (0.04–1.42)	16.06

Each scoring parameter taps into varying spatial processes. 4MT was used as a standard measure of performance to weigh against the ICC of the navigation tasks. *Abbreviations*: VST = Virtual Supermarket test; SHQ = Sea Hero Quest; 4MT = Four mountains Test; ICC = Intra Class Coefficient; CI = Confidence Intervals (lower-upper); MC; Mean change; CoV = Coefficient of Variation. Bold = acceptable test-retest reliability; ICC low test–retest reliability (less than .50); ICC moderate test–retest reliability (between .50–.80); ICC high test–retest reliability (between .80–1.0) according to Koo and Li [35]. The ICC of VST Map drop was not calculated because the administration, scale and marking criteria changed across timepoints.

*Virtual Supermarket Test (VST)*. The VST is a brief measure of path integration, including four tests measures: i). egocentric orientation ii) heading direction iii) allocentric memory and iv) central navigation preference). Two alternative forms were utilised for the current study (paper-based response at T1 and electronic based response at T2). While at T1, a paper version of the supermarket map was used to record responses, an alternative form of the VST was employed 18 months later at T2, to facilitate electronic and automatic recording of participant responses on a 9.7inch iPad. VST trials (1–14) in both versions were identical. At T2, the scoring parameter for VST allocentric memory was updated to include the exact distance of error (here referred to as map drop error), with the specific aim of increasing the sensitivity of the VST sub-measure to measure spatial memory impairment. At T1, participants were categorically given one mark for every response within a 4mm distance of the location target (categorical variable). At T2, participants were marked based on the exact distance of their response from the location target (continuous variable). Subsequently, we did not expect this variable to demonstrate test-retest reliability in the normal range. For a visual representation of the task, please see Coughlan et al. [10] Fig 1: https://www.sciencedirect.com/science/article/abs/pii/S0197458020300348

**Fig 2:**
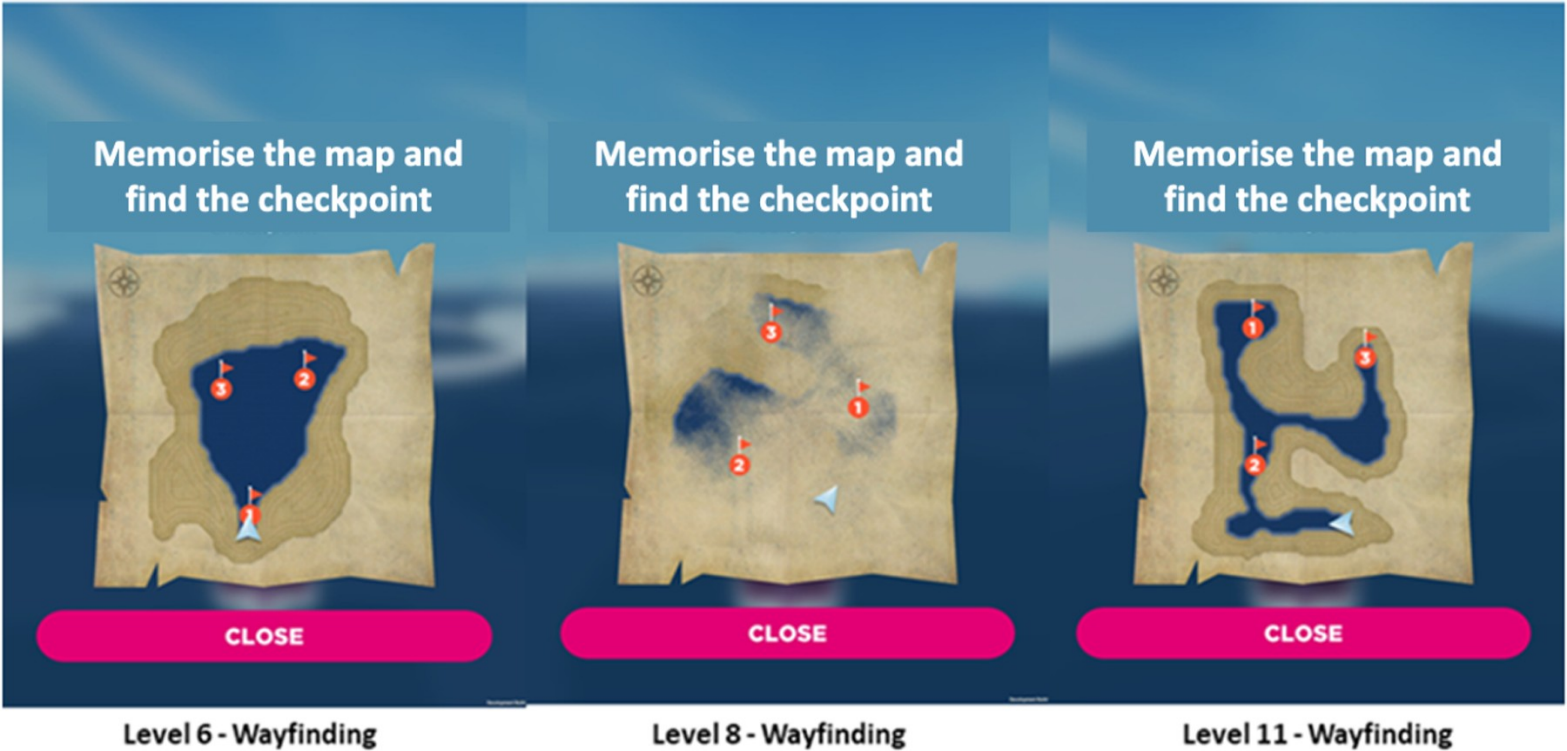
SHQ goal-orientated wayfinding levels (A) 6, (B) 8 and (C). Players initially see a map featuring a start location and several checkpoints (in red) to find in a set order. Checkpoints are buoys with flags marking the checkpoint number. Participants study a map of the level for a recorded number of seconds. When participants exit the map view, they are asked to immediately find the checkpoints (or goals) in the order indicated on the map under timed conditions. As participants navigate the boat through the level, they must keep track of their location using self-motion and environmental landscape cues such as water-land separation. The initiation time is zero as the boat accelerates immediately after the map disappears. If the participant takes more than a set time, an arrow appears pointing in the direction along the Euclidean line to the goal to aid navigation. Adapted from Coughlan et al. [9].

*Sea Hero Quest (SHQ)*. The SHQ game measures path integration through various wayfinding challenges that increase in difficulty over the course of the game. Levels 1 and 2 (motor learning), and levels 6, 8 and 11 (test) were administered. Participants’ i) distance travelled and ii) duration to complete levels is automatically recorded during gameplay and this information is saved on the iPad device in a .json file format (Fig 2). We made the decision not to include an additional SHQ parameter ‘Flare Accuracy’ given limited variation in response (categorical score: 1–3) over only two levels.

*The Four Mountains test*. The electronic version 4MT was included as a standard to measure against the reliability of the novel spatial navigation tasks. The measure taps into short-term allocentric spatial memory. See Chan and colleagues for a full description of the task [24].

### Statistical analyses

#### Neuropsychological performance

We computed linear models with random intercept and time slope per participant to first examine change on neuropsychological test performance over 18 months (change Δ = [retest T2 –baseline T1]). Fixed effects included the *APOE* status and sex. We also included an *APOE* x timepoint interaction term, given ε3ε4 carriers’ greater risk of cognitive decline compared to that of ε3ε3 carriers [32]. In accordance with recommendations, multiple comparisons were not corrected for in the mixed models because separate models were fitted for each performance outcome measure [33]. We report 2-sided *P* values with a significance of .05.

#### Test-retest reliability

To examine the test-retest reliability of each of the three navigation tasks (all include continuous variables) from baseline (T1) to retest (T2), we used 2 complementary approaches:

- Two-way mixed effects intraclass correlation coefficients (ICCs) and 95% confidence intervals according to McGraw and Wong [34]. In addition, the mean difference and the coefficient of variation percentage (CoV%) was computed as an index of measurement variability.
- Repeated measures ANOVAS were used to determine whether effects of *APOE* status or sex contributed to test-retest variability, because ICCs may persist even in the presence of a change over timepoint, or indeed demographic factors such as sex and *APOE* status might influence change. Thus, interactions terms were included in a repeated-measures ANCOVA: APOE × timepoint and sex × timepoint. Including interactions tests for any variance due to an *APOE*/sex × time interaction that unless removed is pooled into the participant × time interaction error variance and inappropriately augments estimated unreliability and biases the ICC downward. This was considered a validation analysis. All scoring parameter listed in Table 3 were the dependent variables. A Bonferroni correction was made to determine the statistical significance of these multiple comparisons in the repeated measures.

## Results

### Neuropsychological performance change analysis

Neuropsychological test performance at baseline and retest are presented in Table 1. There was no significant change in global cognitive performance (Δ) between genetic groups from baseline to retest, except on ACE memory sub-scale (t = 2.41, *p =* 0.02), with ε4 carriers’ performance improving significantly more over the 18-month study period compared to ε3 carriers. The was no significant change on episodic memory concern but change on executive function concern was significantly different between the genetic groups, with ε4 carriers’ showing less increased concern over the 18-month study period compared to ε3 carriers (t = 2.24, *p* = .02). The mean neuropsychological scores across time points, and the mean change across time points, in ε3ε3 and ε3ε4 carriers are also presented in Table 2. At retest, there was no significant difference on ACE performance between genetic groups, with both groups reaching an average performance of 94.37/93.70 out of 100 (see S1 Fig in S1 File).

### Test-retest reliability

Once confirmation that overall global cognitive ability on the ACE and ROCF was intact in the whole sample at the 18-month follow-up, test-retest reliability was measured. For all three test measures and their parameters, intra-class correlation coefficients (model type = mixed) are presented in Table 3. Correlation coefficients ranged from 0.06 (extremely low reliability) to 0.72 (approaching high reliability). Of the seven correlation coefficients presented in each of the measures, five were statistically significantly greater than 0. Three correlation coefficients reflected moderate test–retest reliability (between 0.50–0.80): VST egocentric orientation, VST heading direction, SHQ distance travelled (levels 6,8,11 from the game), and the 4MT total score. The remaining two: the VST central navigation preference and the SHQ game duration parameter reflected low test–retest reliability (less than 0.50). Examination of the CoV% indicated that a number of tasks demonstrated a high degree of variability between the baseline and retest, with many parameters demonstrating a CoV% above 10%. Furthermore, central navigation performance (or CNP) demonstrated a CoV% above 20%. This may be due to the large duration of 18 month between baseline and retest and an alternative form of the VST used between timepoints.

### Validation test–retest reliability based on *APOE* and sex interactions

Repeated measures ANCOVAs specified *APOE* × time interactions and sex × time interactions to test if interactions were biasing the ICC results. One measure approached a significant time × *APOE* interaction: VST central navigation preference (see Table 4 for a summary).

**Table 4:** Mean scores and practice effects on the Virtual Supermarket test, Sea Hero Quest and the Four Mountains test.

Test measure	Mean performance T1	Mean performance T2	APOE	Time	Time × *APOE*	Time × Sex (*p*)
			*p value (F)*	*p value (F)*	*p value (F)*	*p value (F)*
VST ^(n = 56)^						
Egocentric	11.02 ± 3.27	10.16 ± 3.29	.111 (2.628)	.582 (.307)	.327 (.978)	.283 (1.152)
Map drop error	07.53 ± 2.86	234.71 ± 97.21	-	-	-	-
Heading	11.68 ± 2.53	11.76 ± 2.50	.436 (.617)	.145 (2.193)	.876 (.0252)	.576 (.371)
CNP	00.48 ± 0.20	00.43 ± 0.11	.001 (9.021)*	.650 (.209)	.051 (3.552)	.835 (.002)
SHQ ^(n = 44)^						
Distance	4.07 ± .901	3.85 ± .725	.011 (7.040)*	.844 (0.382)	.105 (2.762)	.819 (.178)
Duration	4.96 ± 2.06	4.39 ± 1.31	.298 (1.111)	.670 (.185)	.988 (.003)	.599 (.189)
4MT ^(n = 59)^						
Total	09.76 ± 2.27	10.41 ± 2.18	(.565) .456	.864 (0.301)	.225 (1.505)	.715 (.138)

T1 = baseline, T2 = retest, APOE = apolipoprotein, × = interaction.

None of the measures showed an effect of time in a within-subjects contrast. Between subjects’ contrasts revealed an effect of APOE genotype: VST central navigation performance and SHQ distance. Specifically, ε3ε3 group showed less increase in central navigation preference at retest (baseline: M = .56 ±.21; retest: M = .46 ±.11), than the ε3ε4 group (baseline: M = .38 ± .13; re-rest: M = .40 ±.09). For the SHQ distance parameter, the ε3ε3 group performance remained the same across timepoints (both timepoints: M = 3.77), while the ε3ε4 group’s performance improved from baseline to retest (baseline: M = 4.38 ±.21; retest: M = 3.93 ±.61).

### Navigation at baseline predicts worsening subjective concerns

**Fig 3:**
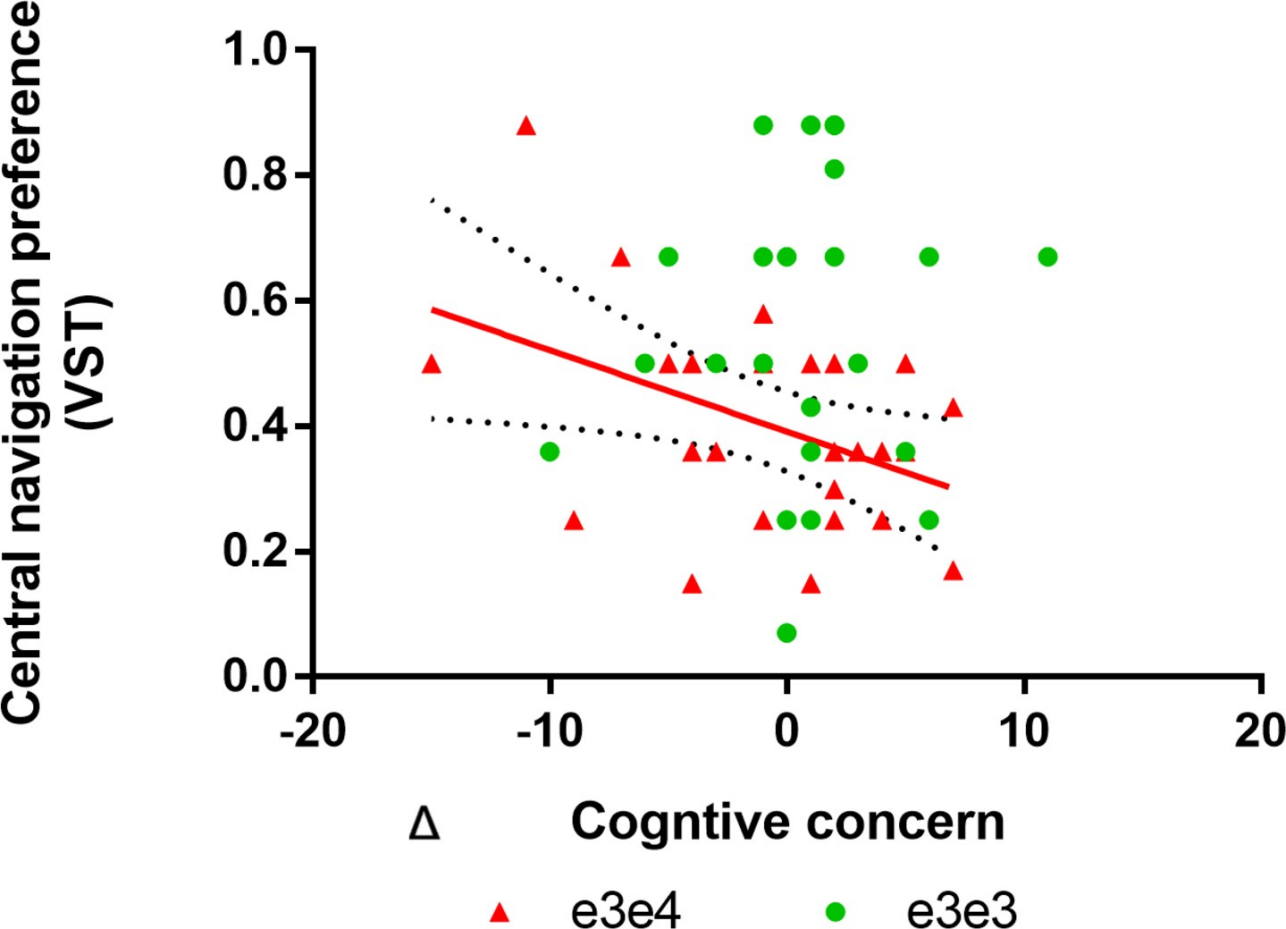
Navigation at baseline predicts worsening subjective concerns. Red line represents a significant association between baseline VST central navigation performance and change on CCI-episodic memory concern in ε3ε4 carriers. Specifically, low baseline central navigation preference (a proxy for boundary-based place memory) predicts increased memory concern increase over 18 months in ε3ε4 carriers. The same association was non-significant in ε3ε3 carriers.

Having determined the test-retest reliability of the novel VST and SHQ measure, as well as the well-established four mountains test measure, we were motivated to examine if the navigation parameters most sensitive to the APOE ε4 at T1, predicted worsening neuropsychological performance or worsening subject cognitive concerns. We took advantage of the baseline analysis at T1, which showed that the entorhinal-PCC mediated VST central navigation preference parameter (a proxy for more boundary-based navigation) distinguishes 73% of high-genetic risk and low-genetic-risk carriers. We used a mixed effects model with an APOE × VST baseline navigation performance interaction term specified. This produced a significant interaction on Δ CCI episodic memory concern (F = 5.07, *P =* 0.02) as the outcome measure, but not on Δ CCI executive function as the outcome measure. Independent models for each genetic group were then specified, revealing that less VST central navigation preference at T1 predicted worsening episodic memory concern in the ε3ε4 carriers (F = 5.01, *P =* 0.03), but not in ε3ε3 carriers (F = 0.15, *P =* 0.69; Fig 3). There was no significant APOE × baseline central navigation preference performance interaction effects on the Δ ACE or ROCF parameters. For results on the cross-sectional effect of *APOE* on the VST and SHQ at retest, please refer to the supplementary results (S2 Fig; S1 and S2 Tables in S1 File) with the inclusion of size participants not tested at baseline.

## Discussion

This study demonstrates the feasibility of implementing novel spatial navigation tests in upcoming randomised control trials as reliable and sensitive preclinical AD markers. Test-retest reliability was assessed in participants, who underwent a retest 18 months following baseline testing. Spatial navigation tests were sensitive for preclinical AD and exhibited moderate test–retest reliability in a nonclinical sample, with some scoring parameters being more reliable than others. Specifically, the VST test–retest reliability correlation coefficients showed the highest test–retest reliability. Three navigation test parameters showed moderate test–retest reliability (VST egocentric orientation; VST heading direction; SHQ distance travelled and the 4MT total score). The remaining three parameters showed low test–retest reliability (VST map drop, VST boundary-based place memory and SHQ duration).

Inconsistent with predictions, there were no APOE genotype or sex interactions with time, suggesting that APOE genotype and sex do not affect the reliability for the tested navigation parameters. However, the APOE interaction with time on central navigation preference (or boundary-based place memory) did trend towards significance. While ε4 carriers remained stable across timepoints, ε3ε3 carriers performed worse at T2 compared to T1. This may indicate that participants actually use a different neural processing sequence at T2 and T1, due to changes made in the administration of the task measure from paper to computerized recording of the allocentric location responses. Thus, the neural correlates of the test measure at T2 should be investigated to look for consistency with neural correlates at T1. Supplementary analysis showed that although the boundary-based navigation measure was less sensitive to the *APOE* genotype at T2 compared to T1, ε4 carriers still travelled a further distance in SHQ relative to non-carriers at T2. Supplementary analysis also showed that time to complete initial SHQ assessment (i.e. SHQ duration) was the greatest predictor of change overtime. Therefore, how long participants initially get to grips with the dynamic environments, such as that in SHQ and VST, should be considered in future longitudinal tracking studies involving these navigation measures. Whether time to complete the initial SHQ assessment will affect the test-re-reliability of the task remains to be established.

Despite different forms of VST administered at both timepoints, the egocentric orientation parameter demonstrated moderate-to-high test-retest reliability, suggesting that this VST parameter translated well from the original form (at T1) to the fully electronic response form (at T2). The SHQ distance travelled measure also demonstrated moderate test-retest reliability. However, post-hoc analysis showed that the effect of *APOE* on retest performance was not replicated, suggesting score stability may not be entirely consistent across timepoints for VST egocentric orientation and SHQ distance travelled, despite reliability across timepoints. This might be due to regression to the mean, which occurs when participants in the lowest quartile of cognitive performance at baseline improve more at retest, compared to participants in the moderate to high quartile of cognitive performance [36]. The *APOE* ε4 effect on both these measures at baseline may be partially driven by novelty effects such that, as a result of initial experience taking the test measure, the newness or novelty of that test disappears the second time, resulting in a small effect of *APOE* at retest. This would also explain why ε4 carriers appear to improve on two of the neuropsychology parameters: ACE memory and CCI executive function.

In similar cognitive studies, Goldberg and colleagues highlighted how practice/novelty effects reduce effect sizes at retest and comprise the utility of preclinical AD test batteries to detect a signal of treatment effect or efficacy in randomized controlled trials [36]. The smaller *APOE* effect on boundary-based navigation measures (VST central preference and SHQ distance travelled) at retest may also have a neural mechanistic explanation. Boundary correction that drives the effect as previously discussed by Kunz and colleagues (2015) is relevant in unfamiliar novel environments primarily [37, 38]. Thus, at retest, the novelty of the environment is lost, and thus grid cell organisations no longer require border cells input if there is time two exposure to the same environment. This may explain why over both timepoints, the risk groups’ grid code dependency on border cell input appears to lessen but not entirely dissipate.

For the VST map drop error parameter (a test of allocentric spatial memory), the individuals’ mean scores changed significantly from the first to the second session. This was expected, as responses were recorded and scored differently at T1 and T2. The original allocentric measure used in T1 described by Tu and colleagues is sensitive, but not specific for AD type dementia [39]. Therefore, the scoring method was altered to capture more AD-sensitive drop placement error for allocentric memory of location responses. Although the mean drop error was larger in the ε4 carrier group compared to the non-carrier group at T2 (which suggests more dispersed allocentric responses), this did not reach statistical significance (see S1 File).

Despite our best efforts to manage regression to the mean at retest by careful selection of statistical methods and alternative forms of VST testing materials between timepoints, there are other statistical approaches to the problem of practice effects. For example, the reliable change index yields information on the number of participants in the sample who demonstrate improvement above and beyond practice. A confidence interval identifies the extent to which an individual participant would have to improve to demonstrate progress beyond a practice effect and beyond all reasonable doubt [40]. Thus, this approach estimates the magnitude of change that exceeds the practice effect and could be explored in future studies.

In terms of the longitudinal analysis, we found very limited evidence of deteriorating cognition in the ε4 carrier group over 18 months. This was expected as it takes up to a 12 years of amyloid/tau accumulation for symptoms of prodromal AD or MCI to onset [41–44]. If AD pathology is indeed present in a proportion of midlife ε4 carriers who displayed disorientation at baseline, pathology would have not spread a significant amount throughout the 18-months. Our preliminary evidence does suggest that more boundary-based place memory on the VST predicts increasing memory concerns over the 18-month period in adult ε4 carriers only. Thus, boundary-based place memory in genetically vulnerable individuals may be predictive of worsening subjective memory concern. This is a significant finding, given that in cognitively intact individuals with elevated amyloid (aged +70 years), subjective cognitive complaints predict global cognitive decline over a 4 year period [45]. Future studies should examine whether *APOE* ε4, in combination with entorhinal-mediated disorientation, predicts dementia risk or prodromal onset in mid to late life adults.

The primary aim of this study was to establish the test-retest reliability of a novel test battery as a sensitive diagnostic and treatment outcome measure for use in preclinical AD studies and RCTs. The VST egocentric orientation and SHQ distance travelled test parameters demonstrated sufficient reliability. Our post-hoc analysis suggests that a combination of genetic (*APOE*) and cognitive (spatial navigation) information predicts worsening episodic memory concern over 18 months. Although boundary-based place memory may indeed be indicative of worsening subjective memory decline in adults at genetic at risk of AD, this parameters’ utility will need to be further investigated before a recommendation for use in clinical and research trials can be made due to its low test-retest reliability score. Further research in larger samples is desirable to ensure that the APOE sensitive navigation parameters meet all the quality metrics for clinical outcome measures.

## Supporting information

S1 File (DOCX)
