# Supplementary material for: Test-retest reliability of spatial navigation in adults at-risk of Alzheimer’s disease
Source: PLoS One. 2020 Sep 22;15(9):e0239077. doi: 10.1371/journal.pone.0239077 (PMC7508365; doi:10.1371/journal.pone.0239077)

**SUPPLEMENTARY INFORMATION**

**SUPPLEMENTARY METHODS**

**Participants**

Exclusion criteria was assessed via telephone screening at baseline. Exclusion factors included psychiatric or neurological disease, substance dependence disorder and clinical depression and/or anxiety. Individuals with medicated cholesterol or blood pressure were included in the study, but medication intake was recorded to ensure there were no differences between genetic groups. Only participants with normal or corrected-to-normal vision were retained due to the nature of the virtual reality tasks. Family history of Alzheimer’s disease (AD) was not included in the analysis, as there was considerable uncertainty when participants were asked the number of parents (0, 1 or 2) with dementia and in particular the type of dementia. Finally, saliva samples were collected via buccal swab from participants who passed this screening and APOE genotype status was determined.

One participant did not complete VST at baseline and four participants did not complete SHQ at baseline. Thus, the cross-sectional comparisons for the effect of APOE on task performance (presented in results below) includes size additional participants.

**SUPPLEMENTARY RESULTS**

**S1 Fig. Follow-up performance on the Addenbrooke’s cognitive examination between genetic groups.** No significant differences between genetic groups on the total score for the Addenbrooke’s cognitive examination at retest (T2).

**
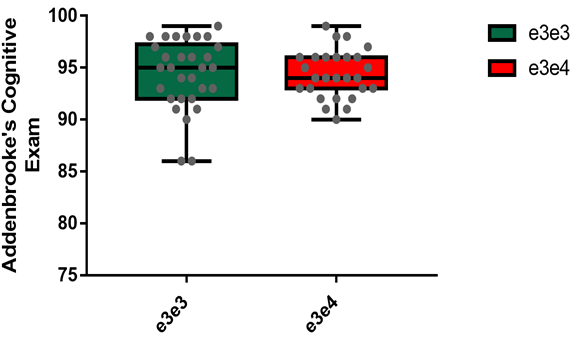
**

**The effect of APOE on the Virtual Supermarket Task and Sea Hero Quest at retest**

We sought examine if there were APOE effects on two VST parameters: Central navigation preference and the allocentric map drop error. The latter sub-measure was introduced at retest (T2). We adopted linear mixed effects models to account for inter-subject variability. Using a mixed model approach, with APOE and sex as a fixed effects and subject as the random effect, there an effect of APOE on VST central navigation preference (t=-2.012, P=.03) with the ε3ε4 group (M=.465, SD=.11) displaying higher boundary-based place memory than the ε3ε3 group (M=.402, SD=.09) (Supplementary figure 2B), reflecting a successful replication of the baseline effect. There was no significant difference between genetic groups on the VST map drop error parameter, which was introduced in T2 only, although there was a trend toward significance with ε4 carriers showing more dispersed allocentric responses than ε3 carriers (Supplementary figure 2A). Table 4 presents the mean scores on both VST measures at T2. There was no main effect of APOE on SHQ performance at T2, but the mean group values did indicate that ε3ε3 carriers outperformed ε3ε4 carriers by travelling a lesser distance (S2 Fig).

**S1 Table**. **Differences between genetic groups on VST performance at retest.** CNP=Central navigation preference.

| VST ^(n=57)^ | *Parameter* | *ε3ε3 (Mean ± SD)* | *ε3ε4 (Mean ± SD)* | *p value* (t value) |
| --- | --- | --- | --- | --- |
|  | Drop error | 219.64 ± 92.33 | 246.41 ± 99.79 | .09 (t=2.97) |
|  | CNP | .465 ± .11 | .402 ± .09 | .03 (t=-2.01) |

**S2 Table. Differences between genetic groups on Sea Hero Quest at retest.** * SHQ levels newly introduced to the test battery at retest

| *Test measure* | *T2 Measure* | *ε3ε3 Mean ± SD* | *ε3ε4 Mean ± SD* | *p value* (t value) |
| --- | --- | --- | --- | --- |
| SHQ ^(n=52)^ | Distance level 6 | .58 ± .16 | .60 ± .11 | *ns* |
|  | Distance level 8 | 1.30 ± .26 | 1.42 ± .34 | *ns* |
|  | Distance level 11 | 1.89 ± 1.94 | 1.97 ± 2.03 | *ns* |
|  | *Distance level 7 | .95 ± .31 | 1.01 ± .32 | *ns* |
|  | *Distance level 21 | 2.59 ± 1.1 | 2.72 ± .86 | *ns* |

The APOE effect on SHQ distance travelled on each level was not significant at retest.

Thus, we predicted that how participants initially got to grips with the task (i.e. the time to complete initial T1 assessment) predicts change on the performance measure of interest: distance travelled. Thus, we entered SHQ baseline duration as a predictor in a general linear model with distance travelled as the outcome variable. T1 duration scores predicted performance change (t=2.65, p=0.01), with higher T1 duration scores at baseline, predicting improved performance on the distance travelled measure. This effect was significant with the inclusion of covariates such as age, sex and APOE genotype status.

**S2 Fig***.* **APOE effects at retest.** A=Allocentric drop error at retest; B=Central navigation preference at retest; C=Distance trajectories on SHQ at retest.


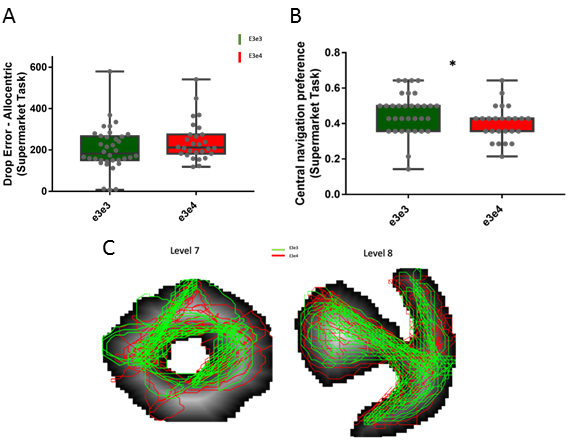

Supplement: S1 File — (DOCX) [file pone.0239077.s001.docx]
